# Supplementary material for: The cryo-EM structure of homotetrameric attachment glycoprotein from langya henipavirus
Source: Nat Commun. 2024 Jan 27;15:812. doi: 10.1038/s41467-024-45202-5 (PMC10821904; doi:10.1038/s41467-024-45202-5)
Supplement: Supplementary file 1 — Supplementary Information [file 41467_2024_45202_MOESM1_ESM.pdf]

Supplementary Information

The cryo-EM structure of homotetrameric attachment glycoprotein from langya henipavirus

Yingying Guo[1,†,*], Songyue Wu[2,3,†], Wenting Li[2,3,†], Haonan Yang[1,†], Tianhao Shi[1], Bin Ju[2,3,*], Zheng Zhang[2,3,*], and Renhong Yan[1,*]

[1]Department of Biochemistry, School of Medicine, Key University Laboratory of Metabolism and Health of Guangdong, Southern University of Science and Technology, Shenzhen 518055, Guangdong Province, China.

[2]Institute for Hepatology, National Clinical Research Center for Infectious Disease, Shenzhen Third People's Hospital, Shenzhen, China.

[3]The Second Affiliated Hospital, School of Medicine, Southern University of Science and Technology, Shenzhen, China.

[†]These authors contributed equally.

*To whom correspondence should be addressed: Yingying Guo (guoyingnba@163.com); Bin Ju (jubin2013@163.com); Zheng Zhang (zhangzheng1975@aliyun.com) or Renhong Yan (yanrh@sustech.edu.cn)

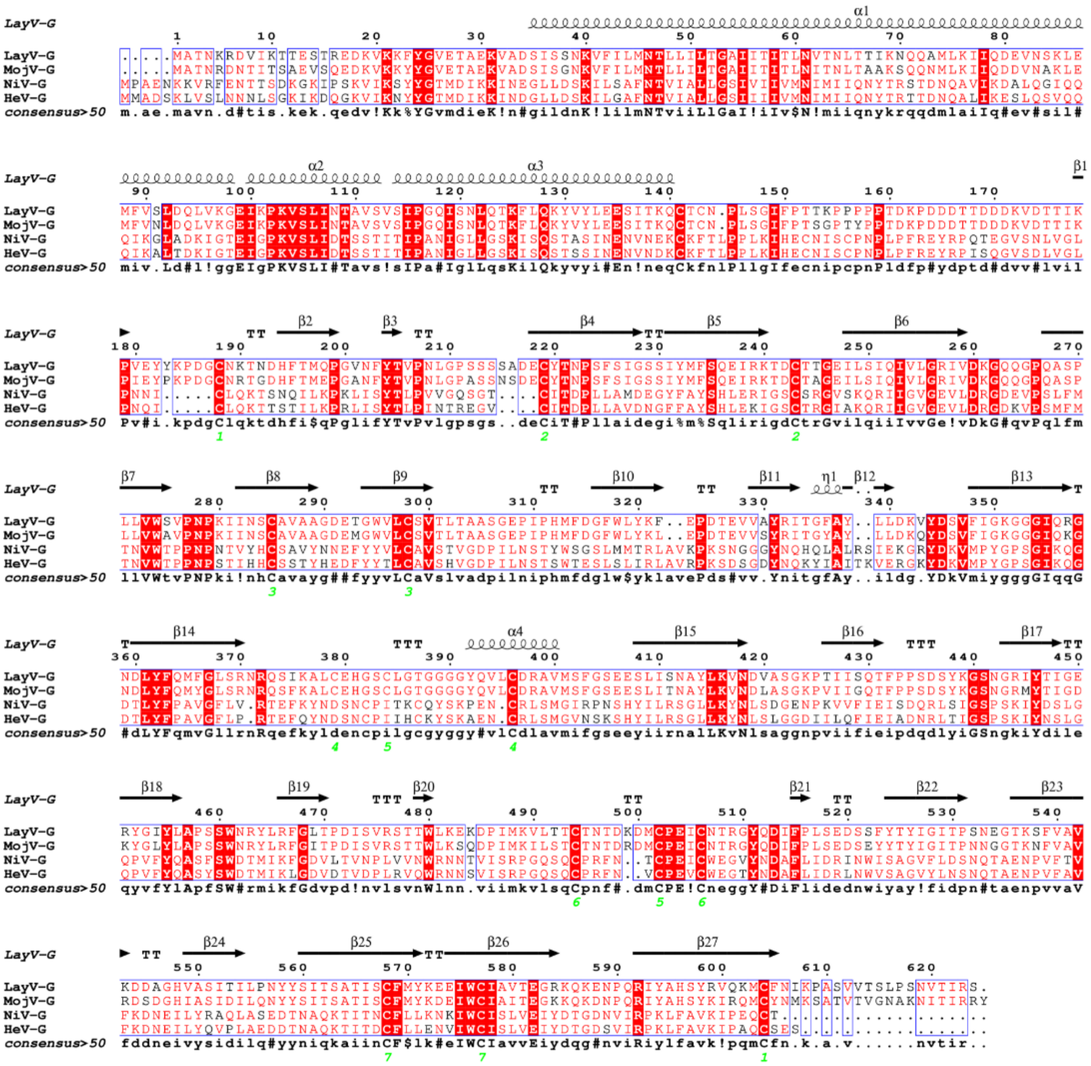

Supplementary Figure 1 Sequence comparison of representative attachment glycoproteins from Henipaviruses.

The sequences including Langya virus (LayV-G, GenBank: UUV47206.1), Mojiang virus (MojV-G, GenBank: YP_009094095.1), Nipah virus (NiV-G, Genbank: NP_112027.1) and Hendra virus (HeV-G, GenBank: NP_047112.2) were aligned using MultAlin 5.4.1 and ESPript 3.0. Cysteine residues involved in disulfide bond formation are indicated with green numbers. The special characters for consensus symbols in the consensus line represent different amino acids: ! is anyone of IV, $ is anyone of LM, % is anyone of FY and # is anyone of NDQEBZ.

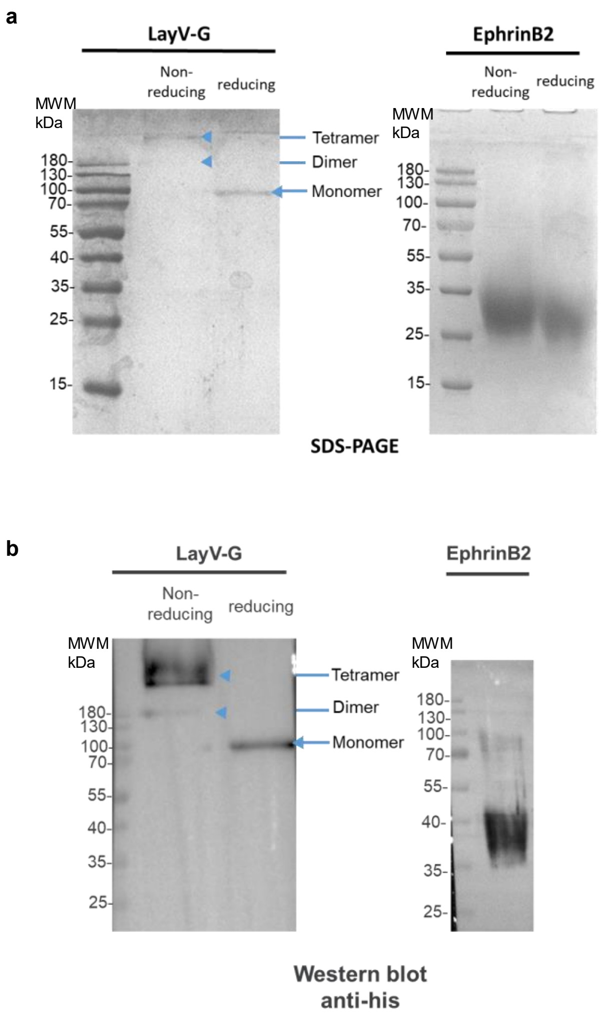

Supplementary Figure 2 Biochemical characterization of the LayV-G and ephrinB2.

a The samples of purified LayV-G and ephrinB2 were subjected to SDS-PAGE under non-reducing and reducing (100mM DTT) conditions to analyze the presence of Tetramer, Dimer, and Monomer. The SDS-PAGE was performed using 12.5% SDS-PAGE and visualized by standard Coomassie brilliant blue staining. **b** Western blot detection performed using anti-His antibodies.

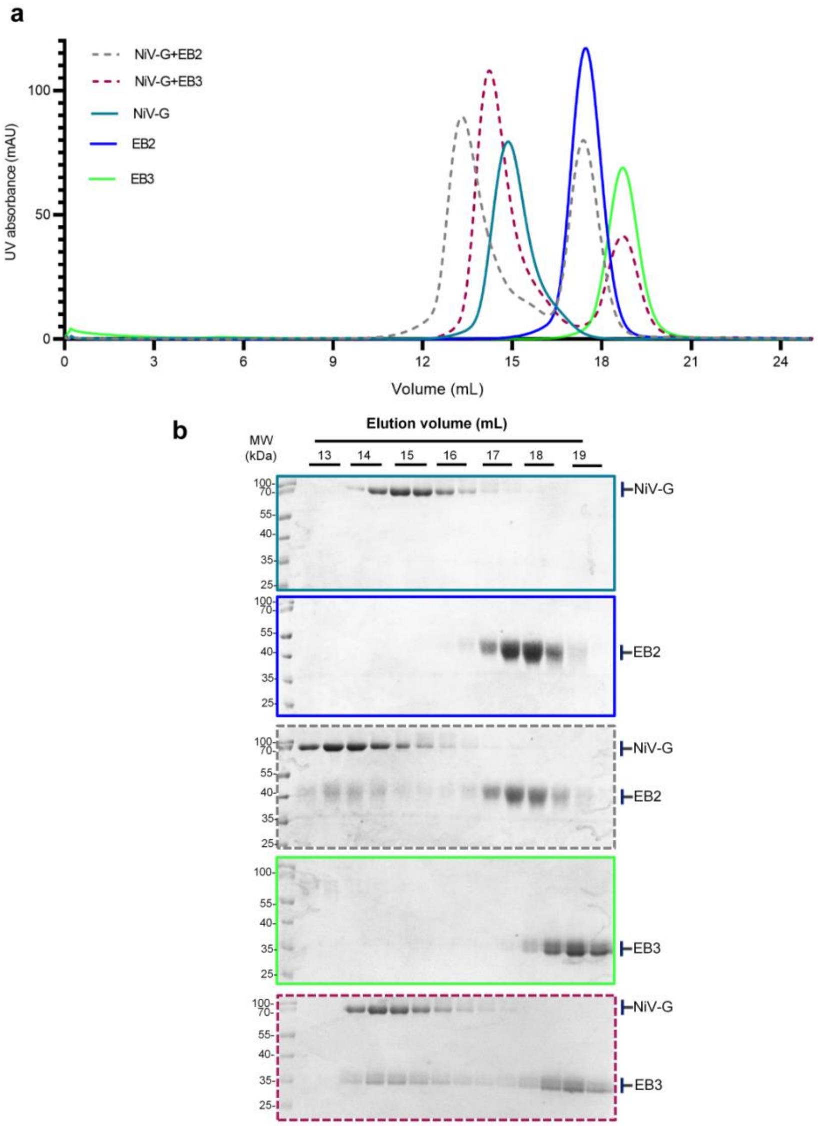

Supplementary Figure 3 Biochemical characterization of the NiV-G binding to ephrinB2 and ephrinB3.

a An incubation of NiV-G and Ephrin-B2/B3, followed by passage through size-exclusion chromatography (SEC) , NiV-G incubated with Ephrin-B2(Silver dotted line) and Ephrin-B3 (Purple dotted line) exhibiting a shift in the peak position of the NiV-G alone (cyan solid line). **b** The samples obtained from size-exclusion chromatography (SEC) collected between 13 mL and 19 mL were subjected to analysis via SDS-PAGE under reducing conditions, Showing the formation of a complex between NiV-G and EphrinB2/B3. EB2: ephrinB2; EB3: ephrinB3. Source data are provided as a Source Data file.

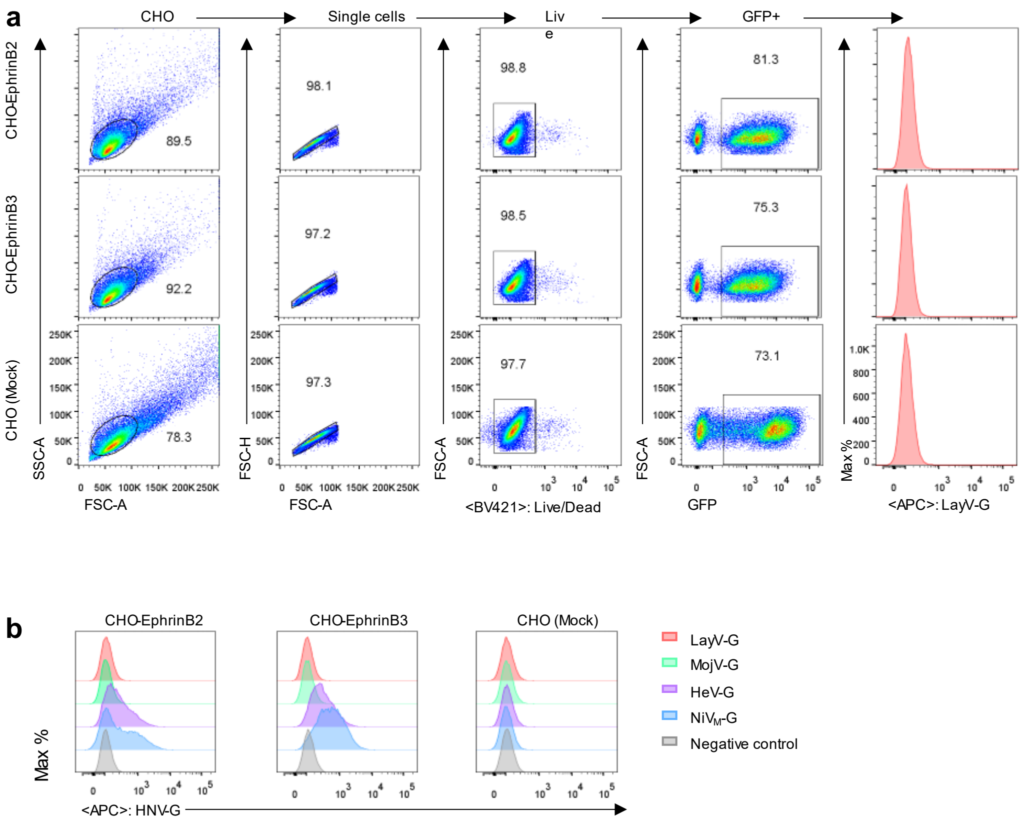

Supplementary Figure 4 Soluble LayV-G does not bind to human ephrinB2 or ephrinB3 expressed on cell surface.

a Full-length ephrinB2, ephrinB3, and mock were separately stably transfected into CHO cells using lentivirus with GFP as a marker to monitor gene expression. The binding of various HNV-G–ECD-His detected by an APC conjugated mouse anti-His monoclonal antibody against the GFP-positive cells were analyzed. **b** Fluorescence intensity histograms show soluble HeV-G and NiV-G but not LayV-G or MojV-G ectodomain with His-tag were able to bind to cell surface ephrinB2 or ephrinB3. No HNV-G protein was added into the incubation in the negative control group. One representative data analysis of two independent experiments is presented here. The mock was stably transfected with empty lentivector with GFP marker. Source data are provided as a Source Data file.

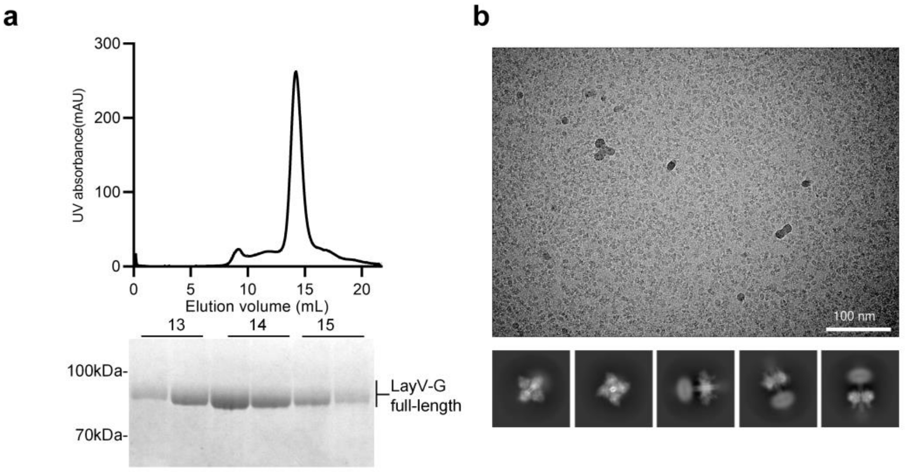

Supplementary Figure 5 Cryo-EM sample purification, micrograph and representative 2D classification.

a The last purification step by size exclusion chromatography (SEC) in the presence of GDN. Source data are provided as a Source Data file. **b** A representative micrograph and 2D class average images are displayed.

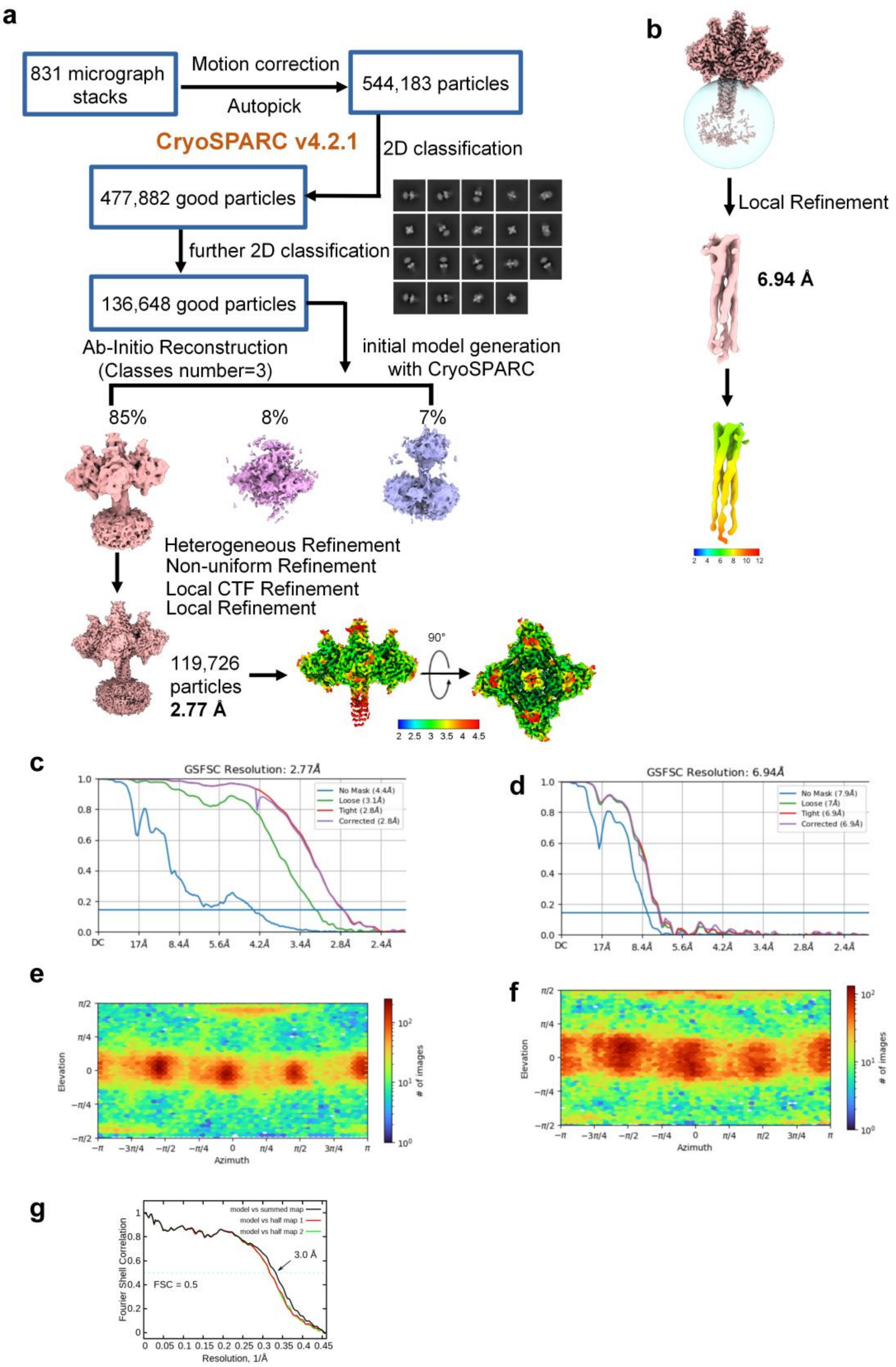

Supplementary Figure 6 Cryo-EM data processing and analysis of LayV-G.

a CryoEM data processing flow chart computed using cryoSPARC 3.3.1 and Estimation of local resolution of the final cryo-EM map. **b** Local refinement of TM domain results. **c,d** The final whole map had GSFSC 0.143 resolution of 2.77 Å, and TM domain local refinement map had GSFSC 0.143 resolution of 6.94 Å. **e,f** Angular distribution of the LayV-G particles in the final round of 3D refinement using

cryoSPARC 3.3.1. **g** Model-map FSC plots calculated by Phenix 1.11.1 between refined map and the atomic model.

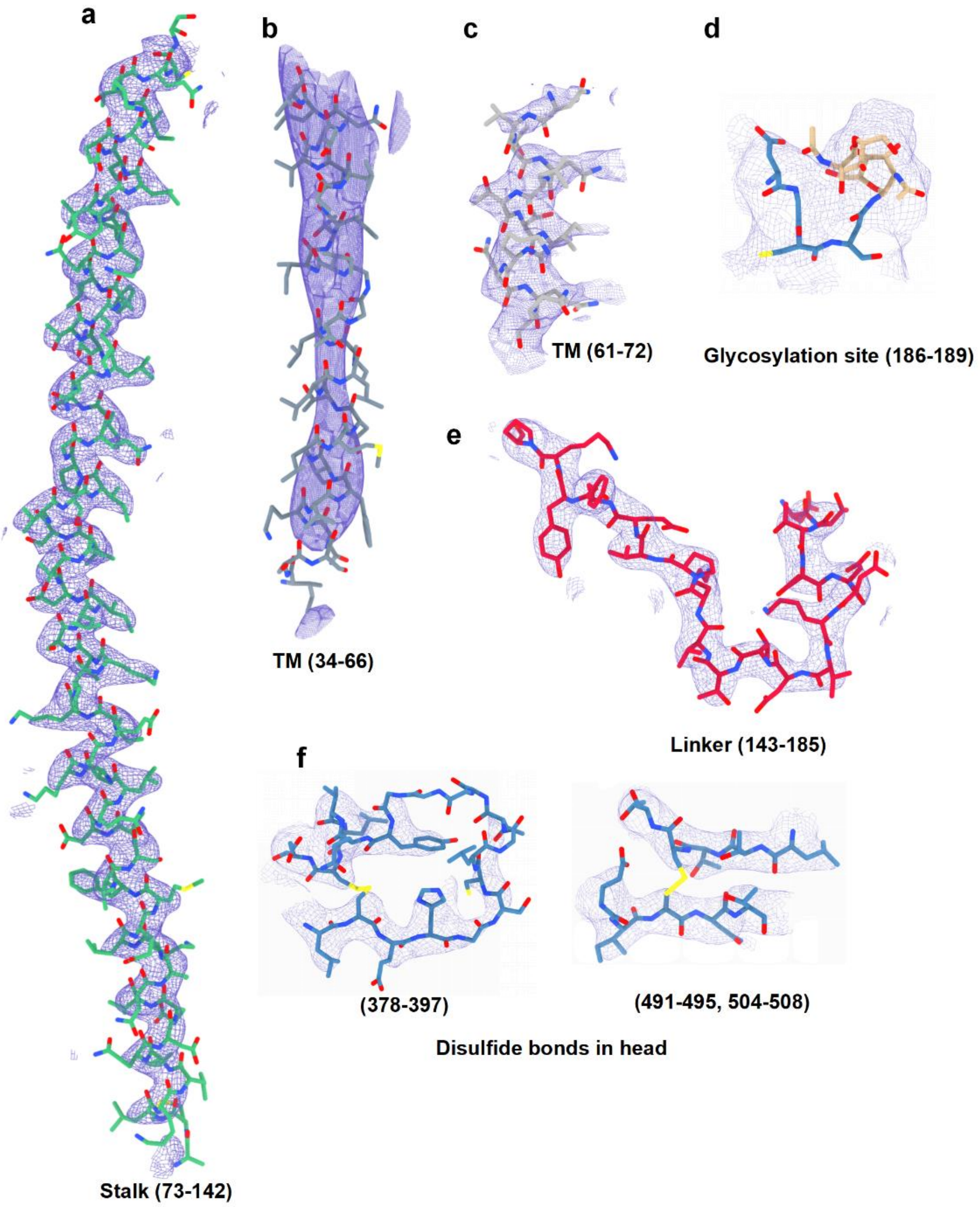

Supplementary Figure 7 Representative cryo-EM density maps of LayV-G.

a Cryo-EM density maps for stalk are shown at threshold of 6 σ. **b** Cryo-EM density map of local refinement TM domain is shown at threshold of 5 σ. **c** Cryo-EM density map of partial TM domain of whole map is shown at threshold of 5 σ. **d** Cryo-EM density maps of glycosylation site is shown at threshold of 7 σ. **e** Cryo-EM density maps of linker is shown at threshold of 6 σ. **f** Cryo-EM density maps of two typical disulfide bonds are shown at threshold of 7 σ.

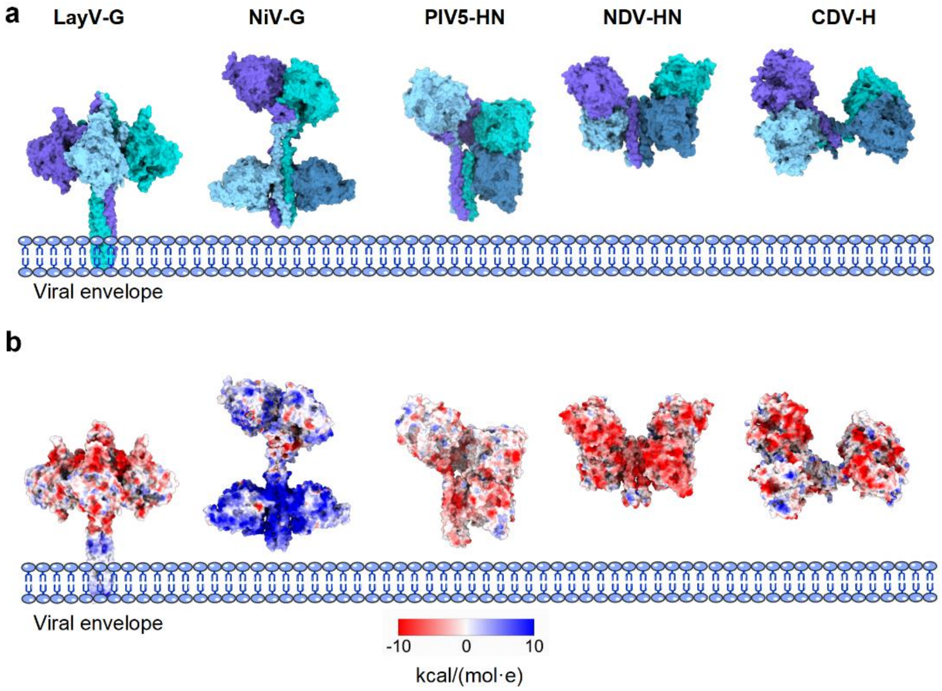

Supplementary Figure 8 Comparative structural gallery of available paramyxovirus attachment glycoprotein architectures.

Five structures including LayV-G, NiV-G (PDB ID: 7TY0 and 7TXZ), PIV5-HN (PDB ID: 4JF7), NDV-HN (PDB ID: 3T1E) and CDV-H (PDB ID: 7ZNY) are near vertical inserted into viral membrane. **a** Structure surfaces are colored by chains according to LayV-G. **b** Structure surfaces are colored by electrostatic potentials, which were estimated in ChimeraX 1.6.1 using coulombic calculation method with default coloring ranging from red for negative potential through white to blue for positive potential. Abbreviations: NiV (Nipah virus), PIV5 (parainfluenza virus 5), NDV (Newcastle disease virus) and CDV (canine distemper virus).

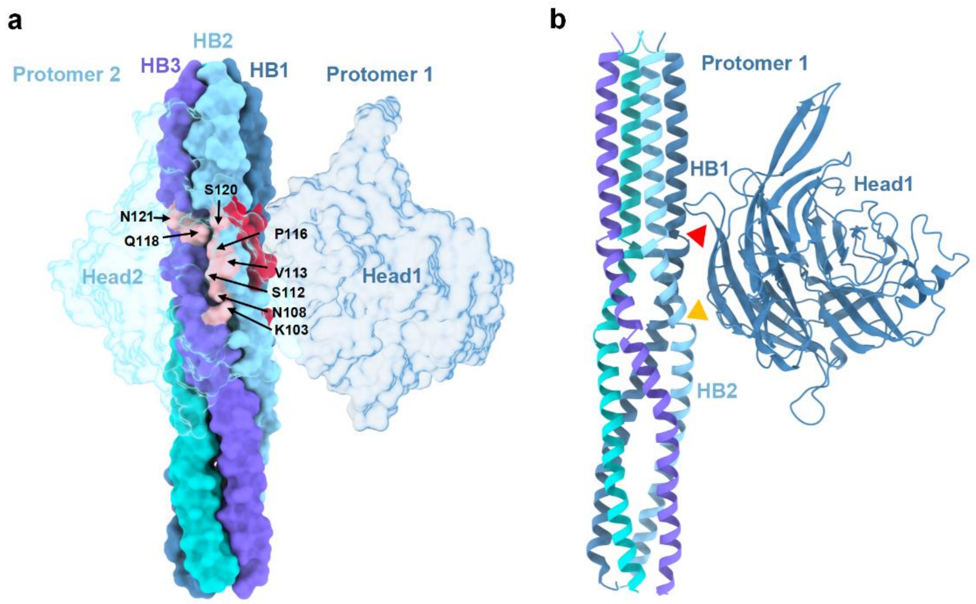

Supplementary Figure 9 The interaction between head and 4HB of LayV-G cause the helices of stalk bend and twist.

a Each head contact with two adjacent 4HB subunits. The head1 interact with the red area from HB1 and HB2, and the head2 interact with the pink area from HB2 and HB3.

b Head1 binding cause two places (yellow triangle and red triangle) of HB1 and HB2 bend and twist, respectively.

Supplementary Table S1. Cryo-EM data collection, refinement, and validation statistics

**Data collection**	
EM equipment	Titan Krios (Thermo Fisher Scientific)
Voltage (kV)	300
Detector	Gatan K3 Summit
Energy filter	Gatan GIF Quantum, 20 eV slit
Pixel size (Å)	1.095
Electron dose (e-/Å2)	50
Defocus range (μm)	-1.4 ~ -1.8
Sample	LayV-G
Number of collected micrographs	831
**3D Reconstruction**	
Software	cryoSPARC 3.3.1
Number of used particles (Overall)	119,726
Resolution (Å)	2.77
Symmetry	C1
Map sharpening B-factor (Å2)	-98.6
**Refinement**	
Software	Phenix 1.11.1
Cell dimensions	
a=b=c (Å)	280.3
α=β=γ (˚)	90
Model composition	
Protein residues	526
Side chains assigned	526
Na	6
Zn	1
Sugar	4
R.m.s deviations	
Bonds length (Å)	0.022
Bonds Angle (˚)	1.266
Ramachandran plot statistics (%)	
Preferred	95.82
Allowed	3.99
Outlier	0.19
**Data availability**	
PDB code	8JZB
EMDB code	EMD-36741

Original gel image

Fig 1a

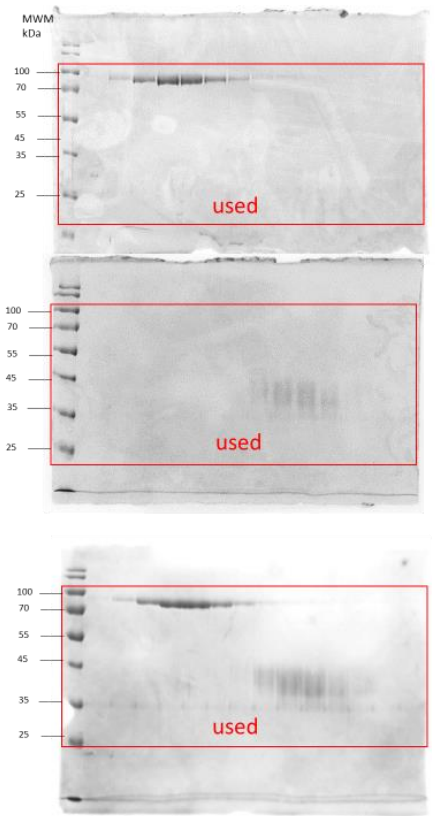

Fig 1b

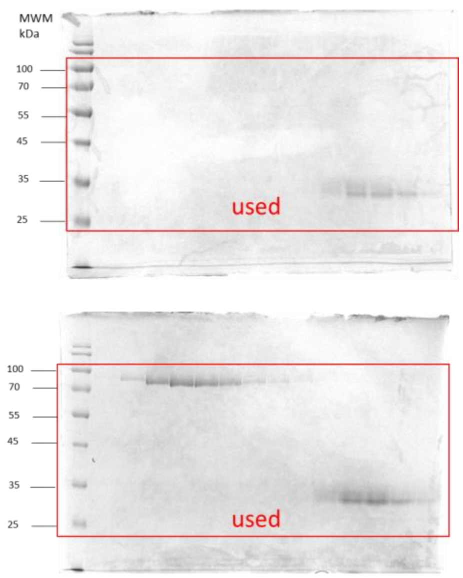

***These gels are shown in Figure 1**

Original gel image

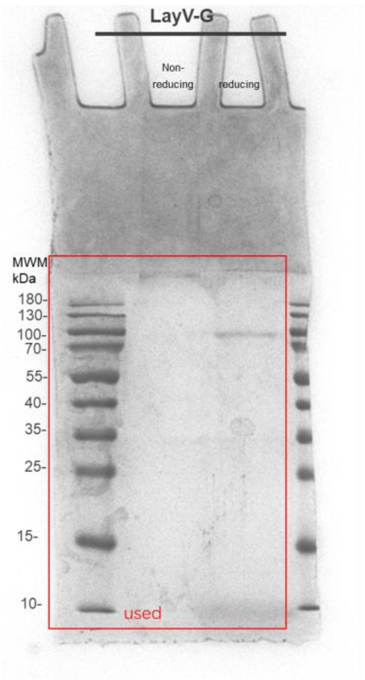

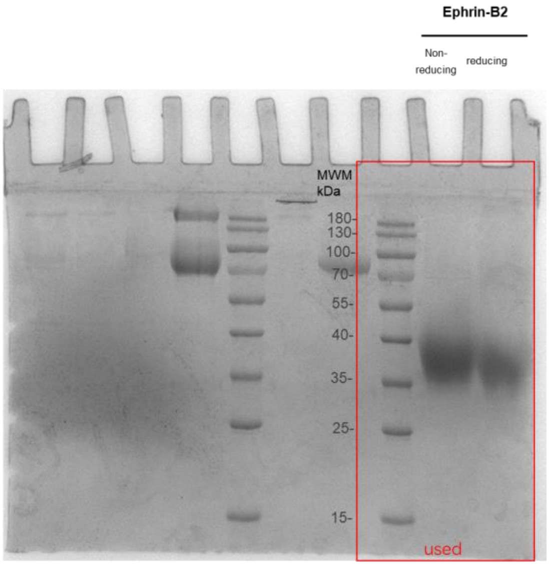

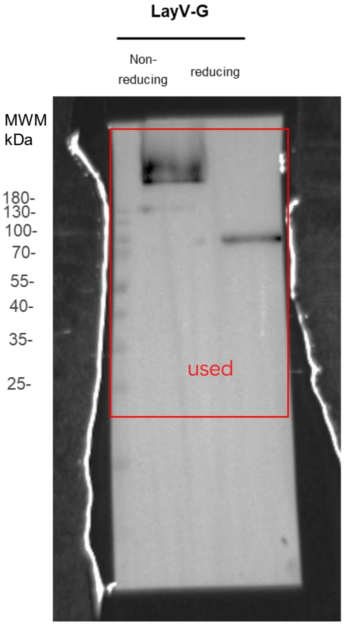

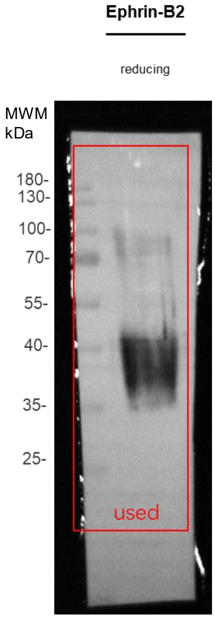

*These gels are shown in Supplementary Fig. 2

Original gel image

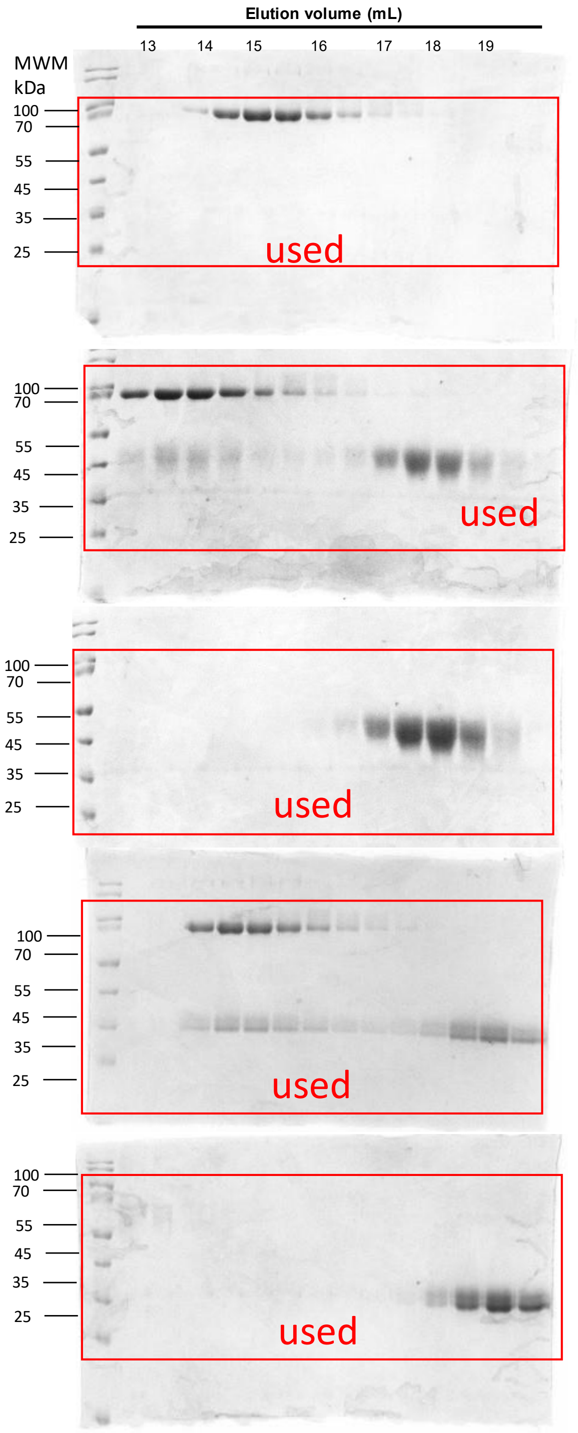

***Theses gels are shown in Supplementary Fig. 3**

Original gel image

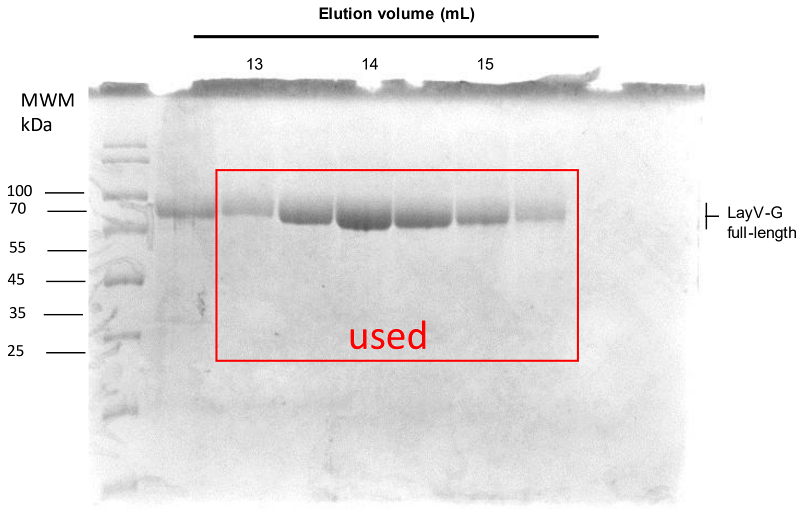

***This gel is shown in Supplementary Fig. 5**
